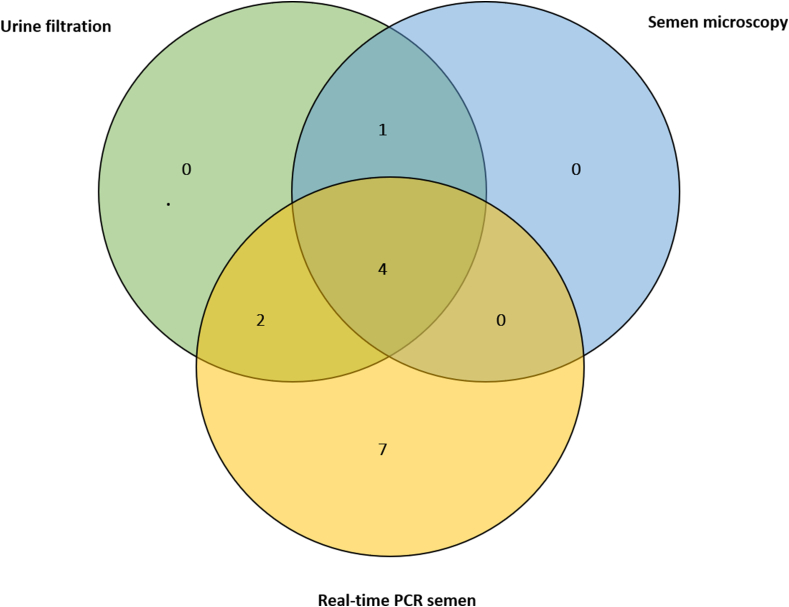# Detection of male genital schistosomiasis (MGS) by real-time TaqMan® PCR analysis of semen from fishermen along the southern shoreline of Lake Malawi

**DOI:** 10.1016/j.heliyon.2023.e17338

**Published:** 2023-06-21

**Authors:** Sekeleghe A. Kayuni, Mohammad H. Alharbi, Alexandra Shaw, Joanna Fawcett, Peter Makaula, Fanuel Lampiao, Lazarus Juziwelo, E. James LaCourse, Jaco J. Verweij, J. Russell Stothard

**Affiliations:** a Department of Tropical Disease Biology, Liverpool School of Tropical Medicine, Liverpool, L3 5QA, United Kingdom; b MASM Medi Clinics Limited, Medical Society of Malawi (MASM), P. O. Box 31659, Lilongwe 3, Malawi; c Malawi Liverpool Wellcome (MLW) Clinical Research Programme, Kamuzu University Of Health Sciences (KUHeS), Queen Elizabeth Central Hospital campus, Chipatala Avenue, Blantyre, Malawi; d Ministry of Health, Buraydah 52367, Saudi Arabia; e Research for Health, Environment and Development (RHED), Mangochi, Malawi; f Physiology Department, College of Medicine, Kamuzu University of Health Sciences, Blantyre, Malawi; g National Schistosomiasis and STH Control Programme, Community Health Sciences Unit, Ministry of Health, Lilongwe, Malawi; h Elisabeth TweeSteden Hospital Tilburg, Microvida Laboratory for Medical Microbiology and Immunology, Hilvarenbeekseweg 60, Tilburg, the Netherlands

**Keywords:** *Schistosoma haematobium*, MGS, Real-time PCR, Fishermen, Mangochi, Lake Malawi

## Abstract

**Background:**

Male genital schistosomiasis (MGS) is an underappreciated complication of schistosomiasis, first described in 1911. However, its epidemiology, diagnostic testing and case management are not well understood in sub-Saharan Africa. To shed new light on MGS prevalence in Malawi, a longitudinal cohort study was conducted among adult fishermen along the southern shoreline of Lake Malawi using detection of schistosome DNA in participants’ semen by real-time TaqMan® PCR analyses.

**Methods:**

Upon recruitment of 376 participants, 210 submitted urine samples and 114 semen samples for parasitological tests. Thereafter, the available semen samples were subsequently analysed by real-time TaqMan® PCR. Praziquantel (PZQ) treatment was provided to all participants with follow-ups attempted at 1, 3, 6 and 12-months’ intervals.

**Results:**

At baseline, real-time PCR detected a higher MGS cohort prevalence of 26.6% (n = 64, Ct-value range: 18.9–37.4), compared to 10.4% by semen microscopy. In total, 21.9% of participants (n = 114) were detected with MGS either by semen microscopy and/or by real-time PCR. Subsequent analyses at 1-, 3-, 6- and 12-month follow-ups indicated variable detection dynamics.

**Conclusions:**

This first application of a molecular method, to detect MGS in sub-Saharan Africa, highlights the need for development of such molecular diagnostic tests which should be affordable and locally accessible. Our investigation also notes the persistence of MGS over a calendar year despite praziquantel treatment.

## 1 Introduction

Male genital schistosomiasis (MGS) is a chronic consequence of urogenital schistosomiasis (UGS), which was first reported in 1911 [1] although is generally underappreciated in nearly all endemic areas [[2], [3], [4]]. Although semen samples are often difficult to obtain, semen microscopy is currently used to visualise schistosome eggs and is considered as a standard detection technique for diagnosis of active MGS infection. Alternatively, urine filtration in conjunction with MGS symptoms is a more convenient diagnostic proxy [5].

As schistosome eggs can be present in semen without their presence in urine, this complicates routine diagnostic surveillance of MGS. This diagnostic challenge contrasts with substantial progress in defining a gold-standard for clinical diagnosis of female genital schistosomiasis (FGS) using colposcopy, and clearly highlights that MGS is more underappreciated than FGS within endemic communities [6].

Moreover, diagnostic sensitivity and specificity of semen microscopy are affected by the diurnal variation of schistosome egg excretions observed in the ejaculate [7]. To avoid these contingencies, molecular techniques such as polymerase chain reaction (PCR) can be applied to MGS [8], which in turn can improve the diagnosis, treatment and monitor the progress of the disease within communities.

We have conducted a longitudinal cohort MGS study among fishermen along the south shoreline of Lake Malawi in Mangochi District, where at baseline, we observed an egg-patent prevalence of 17.1% for UGS and 10.4% for MGS on semen microscopy [5,9]. To better appreciate the dynamics of MGS in Malawi, we applied real-time PCR with TaqMan® probe analysis on collected semen samples to more accurately assess the prevalence of MGS and its dynamics after praziquantel (PZQ) treatment.

## 2 Methods

### 2.1 Study area, population and sampling

Fishermen aged ≥18 years along the south shoreline of Lake Malawi in Mangochi District (Fig. 1) [10] were invited to be recruited into this longitudinal cohort study from October 2017 to December 2018, as previously described [5,9]. Ethical clearance was granted by the National Health Sciences Research Committee (NHSRC) of Malawi (approval number: 1805) and Liverpool School of tropical Medicine Research Ethics Committee (LSTM REC) (approval number: 17–018) to conduct this study, in compliance with all regulations.

**Fig. 1:**
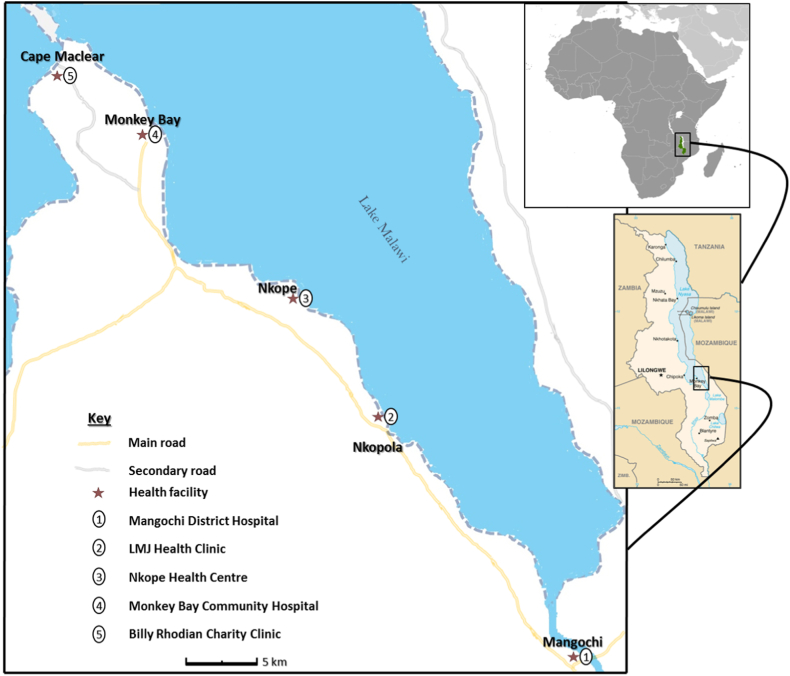
Schematic map of Study area showing health facilities along Lake Malawi in Mangochi district where the study participants were recruited. (*The study map was produced by Dr Sekeleghe Kayuni (4th August 2019)while the maps of Africa and Malawi were reproduced from the maps at the Central Intelligence Agency (CIA) website, public domain:*https://www.cia.gov/library/publications/the-world-factbook/attachments/locator-maps/MI-locator-map.gif*and*https://www.cia.gov/library/publications/the-world-factbook/attachments/maps/MI-map.gif).

### 2.2 Data collection

As described before, the recruited fishermen had questionnaire interviews before being invited to nearby health facilities in the study area to submit urine, stool and semen samples for parasitological analyses, namely urine dipstick, point-of-care circulating cathodic antigen (POC-CCA), filtration, Kato-Katz and semen microscopy ([5,[11], [12], [13], [14]] and in the Supplementary Section).

After microscopy and recording the results per ml of ejaculate [15], the semen was centrifuged, and 0.5 ml of ethanol was added to the semen sediment for preservation before shipment to Elisabeth TweeSteden Hospital (ETZ) in Tilburg, Netherlands for real-time TaqMan® PCR analysis with *Schistosoma*-genus specific DNA probe. The real-time PCR analyses were conducted on semen samples collected from all time-points, as well as on urine samples collected at 6- and 12-months’ time-points (refer to Supplementary Fig. 1).

#### 2.2.1 Real-time TaqMan® PCR analysis with *Schistosoma-*genus specific DNA probe

Ethanol was removed from the preserved semen sediments after centrifugation, as described previously [5]. The pellet was washed twice with phosphate buffered saline (PBS) before suspending in PBS containing 2% polyvinylpolypyrrolidone (PVPP) (Sigma, Steinheim, Germany). The suspension was then heated for 10 min at 95 °C and frozen overnight, before DNA extraction using the QIA symphony Sample Processing (SP) system (Qiagen, Hilden, Germany). In each sample a fixed amount of Phocine Herpes Virus 1 (PhHV-1) was added as an internal control and monitor for inhibition of the real-time PCR, which was performed using primers and probes described previously [16,17]. Amplification, detection, and analysis were performed with the Rotor-Gene Q real-time PCR cycler (Qiagen, Germany).

### 2.3 Statistical analyses

The data collected from parasitological and molecular tests during the study were screened, entered and analysed in Microsoft Excel and SPSS (IBM Statistics 24). Summary statistics were calculated to explore the data and thereafter correlations and significant tests were conducted to describe and interpret the results further using mainly the nonparametric tests.

### 2.4 Ethical considerations

In addition to the ethical clearance and written informed consent, utmost privacy and confidentiality were maintained in the study and all participants were offered PZQ treatment (40 mg/kg) at the end of each visit before invitation for the next follow-up study.

## 3 Results

### 3.1 Study population

Of the 376 recruited participants into the study at baseline, 210 submitted urine (55.9%) and of these, only 114 submitted semen as well (30.3%) [9]. The median age of participants who submitted urine was 30.0 years (interquartile range [IQR]: 15, range: 18–70 years) and for semen was 29.0 years (IQR: 15, range: 18–67 years).

### 3.2 Parasitological testing of urine and semen of the study cohort at baseline

Urine filtration showed that 36 participants (17.1%, n = 210) had *S. haematobium* eggs in urine (UGS) (median egg count: 0.9 per 10 ml; range: 0.1–186.0; IQR: 5.4; volume range: 10–240 ml). Eight (3.8%) were positive for POC-CCA, possibly intestinal schistosomiasis (*S. mansoni*). Twelve participants (10.4%, n = 114) had *S. haematobium* eggs in semen (MGS) (median: 2.9 eggs per ml of ejaculate; range: 0.4–30.0; volume range: 0.1–4.5 ml). Eight participants (66.7%) with MGS had no schistosome eggs in urine. No *S. mansoni* eggs were observed on semen microscopy during the entire study duration.

### 3.3 Real-time TaqMan® PCR for *Schistosoma* DNA

#### 3.3.1 Baseline

**Fig. 2:**
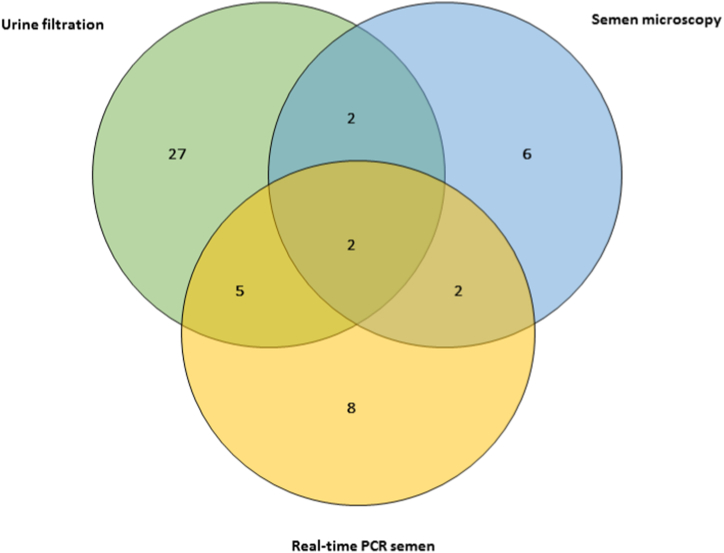
Venn diagram showing positive results of the different diagnostic tests at baseline of the study (n = 52).

Real-time PCR was conducted on 64 semen samples submitted at baseline, due to low volume of some samples. The median age of the participants was 32.5 years (IQR: 22.0), duration of stay was 22.0 years (IQR: 23.8) while their bodyweight was 57.4 kg (IQR: 7.5). Seventeen participants (26.6%) were positive for semen real-time PCR, with median Ct-value of 26.5 (IQR: 8.5, range: 18.9–37.4). Of those MGS participants, eight had no eggs in semen or urine, while six had eggs in semen only. Twenty-seven of those 64 participants who submitted semen samples had eggs in urine only (Fig. 2).

**Fig. 3:**
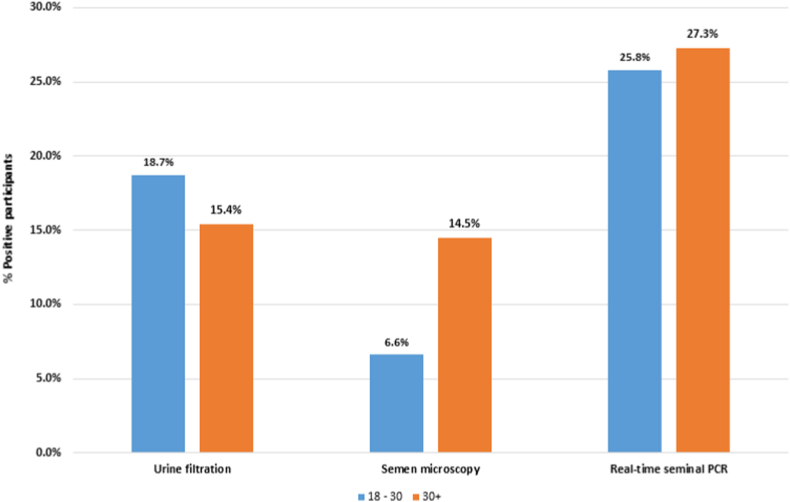
Baseline Bar graph of proportion of positive participants on urine filtration, semen microscopy and real-time PCR per age group (*no statistically significance difference between the age groups*).

For the 114 participants who submitted semen samples, 25 participants were detected with MGS using semen microscopy and real-time PCR, raising the prevalence of MGS from 10.4% to 21.9% (n = 114). Comparing results of all diagnostic tests with age, the proportion of positive semen microscopy and real-time PCR increased with age, as opposed to urine filtration (Fig. 3).

#### 3.3.2 1-Month

Of 114 participants who submitted semen at baseline and invited for follow-up, sixty participants returned and were examined at 1-month follow-up time-point. Real-time PCR was performed on 34 of 41 semen samples submitted and their median age was 34.5 years (IQR: 18.0). While none of those who submitted semen had observed schistosome eggs, nine were positive for real-time PCR, showing MGS prevalence of 26.5%, with median Ct-value of 28.7 (IQR: 9.1, range: 23.7–37.0). Only 3 of the positive participants also had a positive real-time PCR at baseline time-point. Three participants had eggs in urine. Combining semen microscopy and real-time PCR, the prevalence of MGS rose to 29.2%.

**Fig. 4:**
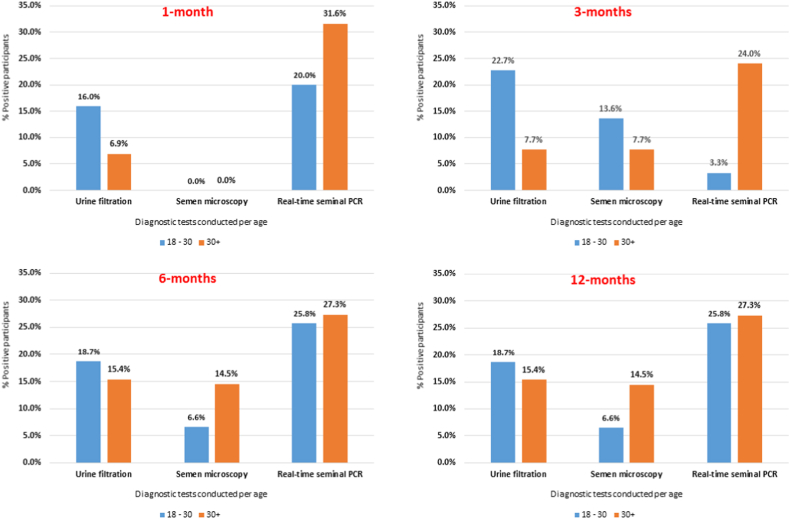
Follow-up Bar graphs of proportions of positive participants on urine filtration, semen microscopy and real-time PCR per age group (*no statistically significance difference between the age groups*).

Six participants (10.5%) had *S. haematobium* eggs in urine (median: 12.8, IQR: 22.9, range: 0.1–29.6). None of the participants were positive for POC-CCA test as well at the other time-points. Looking at all diagnostic tests with age, only the proportion of positive real-time PCR increased with age (Fig. 4).

#### 3.3.3 3-Months

Sixty-four participants were followed at this time-point. Real-time PCR was conducted on 46 semen samples submitted from 48 participants and median age was 33.0 years (IQR: 21.0). Thirteen participants (28.3%) were positive for real-time PCR, with median Ct-value of 28.3 (IQR: 8.5, range: 22.5–36.9). Seven participants with positive real-time PCR had no schistosome eggs in semen or urine while four had eggs in both urine and semen (refer to Supplementary Fig. 2). Six of the positive semen real-time PCR participants also had positive result at baseline of which 4 were also positive at 1-month time-point, with only 2 being positive at all the three time-points. Using semen microscopy and real-time PCR, MGS was detected in 14 participants, showing a prevalence of 29.2% (n = 48).

Seven participants (14.6%) had *S. haematobium* eggs in urine (median: 9.0, IQR: 19.2, range: 0.3–69.0). Comparing the diagnostic tests with age, only proportion of positive participants on urine filtration, semen microscopy and real-time PCR decreased with age (Fig. 4).

#### 3.3.4 6-Months

At this time-point, 63 participants took part in the study and 49 of the semen samples submitted underwent real-time PCR analyses (median age: 31.0 years, IQR: 18.0). Eight participants (16.3%) were positive for real-time PCR, median Ct-value of 31.3 (IQR: 5.0, range: 23.4–36.1) and none had schistosome eggs in urine or semen.

Five of the 8 positive participants were also positive at 3-months’ time-point (of which one was also positive at 1-month) while 2 were positive only at baseline. Ten participants were detected with MGS using semen microscopy and real-time PCR, showing MGS prevalence of 18.9% (n = 53).

Two participants (3.3%) had *S. haematobium* eggs in urine (mean: 1.95), 2 (3.8%) in semen (mean: 0.85). Looking at the diagnostics tests used at this time-point, the proportion of positive participants of all the tests increased with age, as opposed to previous time-points (Fig. 4).

#### 3.3.5 12-Months

Forty-five participants were examined at this time-point, real-time PCR conducted on 41 of 44 semen samples and the median age was 35.0 years (IQR: 21.0). Twelve participants were positive for real-time PCR, showing MGS prevalence of 29.3%, with median Ct-value of 28.4 (IQR: 14.6, range: 17.6–36.6). Of those with positive real-time PCR, 8 had no schistosome eggs, 2 had eggs in urine only, 1 in stool (on Kato-Katz) and another one in semen. In addition, real-time PCR was also conducted on 48 urine samples at this time point, with only 3 (6.3%) urine samples being positive (mean Ct-value: 31.0, SD: 5.43, range: 25.1–35.8).

Four participants (8.3%) had *S. haematobium* eggs in urine (median: 3.05, IQR: 2.8, range: 0.8–4.1). Comparing the results of all diagnostic tests with age, a similar trend to that of 6-months’ follow-up was observed with all the proportions of positive participants decreasing with age (Fig. 4).

#### 3.3.6 Comparison of the different diagnostic tests

When using semen real-time PCR as reference test, the sensitivities of the other diagnostic tests ranged from 14.3% (semen microscopy) to 52.4% (POC-CCA) while the specificities ranged from 67.9% (POC-CCA) to 93.2% (urine filtration) (Table 1). Only urine filtration correlated significantly with real-time PCR, with higher Kappa measure of agreement value (0.31) and stronger statistical significance than other tests (*p* = 0.002).

**Table 1:** Outcomes of the parasitological diagnostic tests compared with semen real-time PCR of the study participants.

Test	Total (N)	Sensitivity (%)	Specificity (%)	Kappa		Spearman correlation		Chi-squared test	
				value	*p-value*	Coefficient (rho)	*p-value*	value	*p-value*
Urine filtration	210	33.3	93.2	0.31	0.002	0.34	0.002	9.21	0.002
Semen microscopy	114	14.3	91.5	0.07	0.45	0.09	0.45	0.58	0.45
POC-CCA (antigen)	210	52.4	67.9	0.18	0.10	0.19	0.11	2.64	0.10
Positive by any parasitological test	210	33.3	84.7	0.20	0.08	0.20	0.08	3.16	0.08
Positive by any parasitological or antigen test	**210**	**71.4**	**59.3**	**0.24**	**0.02**	**0.27**	**0.02**	**5.86**	**0.02**

**Fig. 5:**
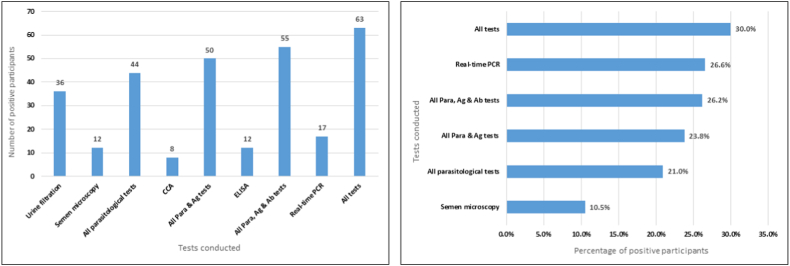
Bar graphs showing study participants who were positive on diagnostic tests at Baseline.

The sensitivity increased while specificity decreased when any positive results from parasitological and antigen tests were compared with real-time PCR results. When results of the diagnostic tests are combined to describe the prevalence of MGS, it shows that prevalence goes up from 10.4% (using semen microscopy only) to 30.0%, as shown in Fig. 5, using results from the baseline time-point.

## 4 Discussion

Since its first description over a century ago by Madden [1], the prevalence of MGS remains largely unknown, unrecognised, misdiagnosed and underreported among men in endemic areas such as shores of Lake Malawi. Coupled with the long known poor health-seeking behaviour of men, MGS can adversely impact the male reproductive health, making it an ignored aspect of an NTD and a public health concern in such endemic areas. To our knowledge, this longitudinal cohort study among local fishermen along the south shoreline of Lake Malawi was the first original research to investigate the prevalence of MGS in a schistosomiasis-endemic area, following several previous case descriptive reports of travellers or non-dwellers visiting the lake for recreation or business [8,18,19]. Our first application of a molecular detection method within a community cohort has now revealed clear shortcomings of parasitological surveillance methods alone.

### 4.1 Diagnostics for UGS with special focus on MGS

The diagnosis of UGS includes inexpensive, urine filtration which suffers low sensitivity and specificity and can be lacking in most endemic areas due to the limited resources and inadequate laboratory capacity, overburdened with other important prevalent diseases like malaria, diarrhoea, pneumonia and HIV. Although semen microscopy is considered as a standard diagnostic method for MGS, perceptions and challenges encountered in semen submission and analysis [20] have resulted in urine filtration being used as a proxy to diagnose MGS. Both methods suffer from the day-to-day diurnal variations in excretion of eggs into urine and semen, affecting their sensitivity and specificity which requires repeated sample submission and examination on consecutive days in order to improve detection of infected people [21].

The present MGS study, comprising of fishermen aged between 18 and 70 years old, who spent most of their life on lake shoreline, observed a 17.1% prevalence for UGS and 10.4% for MGS using semen microscopy at baseline [5]. Also, most MGS participants in the study at baseline (66.7%) did not have schistosome eggs in urine, highlighting the need for more sensitive diagnostic tests such as molecular techniques.

### 4.2 Novel findings with real-time PCR diagnostics for MGS

Development of PCR has revolutionised the field of medicine, improving diagnosis of prevalent diseases especially NTDs. Detection of parasitic DNA in specimens from people exposed to infective parasites in endemic areas confidently indicate presence of the infection, which demonstrates the high sensitivity and specificity of this diagnostic technique and contribute to control, treatment and prevention. Real-time PCR of schistosomiasis has also demonstrated superior diagnostic performance in areas of low transmissions and non-endemic populations [21].

This technique has been applied to various samples including urine, stool and vaginal secretions to detect *Schistosoma* DNA in comparison with traditional standard tests and yielding significant, more informative results in schistosomiasis diagnosis [16,17,22]. However, this molecular technique is quite expensive and not readily available in endemic areas, as in Malawi where reference laboratories do not routinely conduct analyses for detection of schistosome DNA. It is worthy to note that our study is the first original research in an endemic area to apply semen real-time PCR to diagnose MGS, detecting 26.6% of participants at baseline increasing the overall prevalence of MGS by 2-fold.

Conducting other diagnostic tests in addition to urine filtration, such as urine reagent multistix strips and POC-CCA can help in improving the diagnosis of schistosomiasis, identifying other infected people with other *Schistosoma* species [12,21,23] and subsequently MGS. Our study demonstrated a significant Kappa agreement of measurement between any positive parasitological or POC-CCA (antigen) test with semen real-time PCR (*using a genus-specific probe*) as a reference test (*p* = 0.02), correlation (*p* = 0.02) and statistical difference (*p* = 0.02). Also, urine filtration showed low sensitivity of 33.3%, high specificity of 93.4%, a significant Kappa agreement of measurement (*p* = 0.002), correlation (*p* = 0.002) and statistical difference (*p* = 0.002). Overall, the number of people with schistosomiasis was seen to increase with multiple diagnostic methods, showing a higher prevalence of schistosomiasis than individual method.

At the baseline of the study, eight of 17 participants who were positive on semen real-time PCR had no eggs in urine or semen which highlights the need for multiple diagnostic assays as well as using more sensitive tests to detect more cases of MGS. Also, real-time PCR did not pick up all MGS cases as observed in participants with semen eggs but negative on PCR, which could explain among diagnostic challenges, an old infection with possibly calcified eggs migrating in the genital tissues and then released into ejaculatory ducts and seminal fluid, resulting in lack of PCR signal.

Looking at the trend of the tests with age, the proportion of MGS participants diagnosed by semen microscopy and real-time PCR was noted to be increasing with age, compared to urine filtration. This could be due to MGS developing later in life as a result of repeated exposure to infested water bodies, having re-infections [24,25] and presenting as a chronic consequence of schistosomiasis, especially UGS [18].

However, there have been reports of MGS at younger age [26,27], showing that since schistosomiasis starts as early as infancy [28], MGS can develop even before adolescence, as the children have frequent exposure to infested waters, experience high re-infection rates despite at times being provided PZQ treatment which has good cure rates and reverses early pathologies [29]. As part of this study, PZQ was provided during the study and clearance of MGS was assessed among the participants.

### 4.3 Efficacy of standard single-dose PZQ treatment on progression of MGS

Following the MGS participants after PZQ treatment, the study showed that schistosome eggs in most participants cleared in semen 1-month later, despite 26.5% of 34 participants tested with real-time PCR being positive. This indicates presence of *Schistosoma* DNA in infected participants who were not excreting eggs in semen and limiting semen microscopy in MGS at this time-point, supporting the low sensitivity of microscopy in diagnosing schistosomiasis. Also, comparison of the trend of diagnostics tests with age was similar as a baseline. At 3-months’ time-point, schistosome eggs were detected in 10.4% of participants’ semen with 28.3% being positive for real-time PCR, increasing the MGS prevalence to 29.2%. This could possibly be resulting from participants reinfected from same infested water bodies after PZQ treatment.

Similarly, at 6- and 12-months, the prevalence of MGS was 18.9% and 29.3% respectively, demonstrating the possible re-infection of the study participants due their repeated exposure to the infested lake waters where they continued to conduct the income-generating fishing activities. This highlights the need for comprehensive control strategies for schistosomiasis such as adequate health education to modify and transform behaviour related to schistosomiasis risk [30], engaging men in water, sanitation and hygiene (WASH) interventions, intermediate snail-host control, in addition to improving access to treatment in local health facilities and to inclusion and participation in PZQ MDA campaigns [31], to reduce the prevalence and complications of MGS.

### 4.4 Limitations

The low number of participants submitting semen samples limit the generalisation of the study results to the male population in the country and endemic region. This could be explained by negative perceptions and myths associated with semen in rural communities, although previous study examining semen in the district did not encounter such challenges [32]. Also, single urine and semen samples submitted by a majority of participants at each time-point may have affected the sensitivity of the results and underestimated the true burden of the disease.

In addition, the rumours going around in the study area of blood suckers (vampires) visiting and perturbing the local communities during study data collection at baseline time-point, negatively affected the trust and confidence on the team to proceed with the study [33], although additional sensitisation and discussions were conducted with local traditional and opinion leaders, health workers and police officers.

Also, some participants were reluctant to submit samples at the health centres, due to poor health-seeking behaviour. However, previous studies describing MGS had similar or even lower number of participants submitting semen, hence our results contribute to the current knowledge of MGS in local inhabitants of a schistosomiasis endemic area.

## 5 Conclusion

Applying more sensitive and specific semen real-time PCR technique improves diagnosis of MGS. In endemic areas like Lake Malawi shoreline, it was a prevalent, unrecognised manifestation of schistosomiasis, the detection of which increased by two-fold by using molecular methods. This highlights a clear need for a more accessible routine molecular diagnostic test alongside parasitological tests within the clinical practice.

This in turn could lead to more timely provision of individual PZQ treatment and considerations in increasing the frequency of PZQ preventive treatments given through MDA campaigns from annually to bi-annually.

## Summary

Male genital schistosomiasis (MGS) remains a poorly recognised consequence of urogenital schistosomiasis owing to limited diagnostic testing in endemic areas. Improving the future availability and accessibility of sensitive tests like real-time polymerase chain reaction (PCR) could contribute significantly towards better descriptive epidemiology and case management of MGS.

## Authors’ contributions

S. A. K., E. J. L., J. J. V. and J. R. S. conceived, designed the study and laboratory tests; S. A. K., M. H. A., A. S., J. F., P. M., E. J. L. and J. R. S. conducted the sample collection in the field; S. A. K., M. H. A., A. S., J. F., J. J. V., E. J. L. and J. R. S. performed the laboratory tests, analysed and interpreted the data; S. A. K., M. H. A., F. L., L. J., J. J. V., E. J. L. and J. R. S. contributed reagents, materials and analysis tools; and all authors wrote the manuscript.

## Funding

S. A. K. was funded by a PhD scholarship from 10.13039/501100000867Commonwealth Scholarship Commission, United Kingdom; an International Travel Fellowship from the British Society for Parasitology; grant from World Friendship Charity; and African Research Network for Neglected Tropical Diseases (ARNTD) through 10.13039/100000200United States Agency for International Development (USAID), UK aid from the British people (UKaid) and Coalition for Operational Research on Neglected Tropical Diseases (COR-NTD) to conduct the longitudinal cohort study on MGS. M. H. A. was funded by a PhD scholarship from Ministry of Health of Kingdom of Saudi Arabia. A. S. and J. F. were supported by 10.13039/100014976LSTM Education Department MSc Degree Research Project Module funding. The contents of this publication are solely the responsibility of the authors and not their funders.

## Data availability statement

Data included in article/supp. material/referenced in article.

## Declaration of competing interest

The authors declare that the research was conducted in the absence of any commercial or financial relationships that could be construed as a potential conflict of interest.

## Figures and Tables

**figs1:**
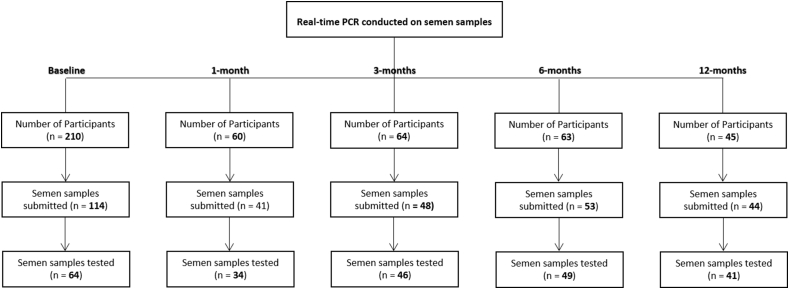


**figs2:**