# Supplementary material for: Detection of male genital schistosomiasis (MGS) by real-time TaqMan® PCR analysis of semen from fishermen along the southern shoreline of Lake Malawi
Source: Heliyon. 2023 Jun 21;9(7):e17338. doi: 10.1016/j.heliyon.2023.e17338 (PMC10394912; doi:10.1016/j.heliyon.2023.e17338)
Supplement: Multimedia component 1 [file mmc1.docx]

**Supplementary Section**

**Description of the Data collection methods**

The following are the data collection methods that were used in the study:

***Parasitological analyses***

The recruited study participants were provided with a clean sample container at the health facility to submit urine, between 10am and 2pm for filtration to examine for schistosome eggs to confirm urogenital schistosomiasis (UGS). Semen was submitted in a clear, transparent, self-sealing plastic bag after abstaining from coitus for two days to examine for male genital schistosomiasis (MGS), defined in the study as the presence of schistosome eggs in semen.

***Urine analysis***

1. ***Visual inspection with urine colour card***

Urine was analysed immediately for macrohematuria by visual inspection using a urine colour card, and then for microhematuria, leukocytes and proteinuria using reagent strips (Siemens multistix 10G) and scores were recorded in the following categories: negative, trace, +, ++ and +++.

1. ***Point-of-care circulating cathodic antigen (POC-CCA)***

Point-of-care circulating cathodic antigen (POC-CCA) test was conducted on the urine to assess for possible intestinal infection by *S. mansoni*, following manufacturer’s instructions (Rapid Medical Diagnostics, South Africa; batch no. 171103130) and as described previously (van Dam et al., 2004). Urine was measured and recorded accordingly, before conducting filtration following approved standard guidelines (WHO, 1991; Cheesbrough, 2009).

1. ***Urine filtration and microscopy***

The entire volume of urine was filtered through a disinfected filter containing a clean polycarbonate membrane with 20 μm pores to trap as many S. haematobium eggs in the sample. The membrane was removed, placed on a standard glass slide and examined under the microscope.

Iodine was added to visualise the eggs distinctly. The number of eggs was calculated by first, dividing the total eggs observed by the total volume filtered and then multiplying by 10. The resultant egg count was recorded per 10 ml of urine. Highest infection intensity for UGS was defined as egg count of ≥ 50 eggs per 10 ml urine as widely described (Cheesbrough, 2009).

***Seminal microscopic analysis***

After submission, the bag with semen was placed under room temperature on a clean bench surface to allow the semen to liquefy. Thereafter, the semen was pushed gently to one corner of the clear plastic bag. Then the bag was heat-sealed to evenly concentrate the semen for easy visualization during microscopy. Direct examination of the semen bag was conducted under a microscope to check for schistosome eggs and the presence of leukocytes (WHO, 2010), thereafter the results were recorded as per ml of ejaculate.

Afterwards, the semen was measured and centrifuged at 3000 *xg* for 5 minutes to collect the seminal plasma. The sediment was re-dissolved in 0.5 ml normal saline for wet mount inspection using 2-3 drops and placed on a slide with a coverslip for microscopy, followed by recording of the results. Thereafter, 0.5 ml of ethanol was added to the remaining sediment for preservation and stored together with the seminal plasma at -80°C in preparation for shipment to the United Kingdom for real-time polymerase chain reaction of *Schistosoma* genus DNA.
