# Supplementary material for: Detection of male genital schistosomiasis (MGS) by real-time TaqMan® PCR analysis of semen from fishermen along the southern shoreline of Lake Malawi
Source: Heliyon. 2023 Jun 21;9(7):e17338. doi: 10.1016/j.heliyon.2023.e17338 (PMC10394912; doi:10.1016/j.heliyon.2023.e17338)
Supplement: Multimedia component 4 [file mmc4.pdf]

Telephone: + 265 789 400
Facsimile: + 265 789 431

All Communications should be addressed to:

The Secretary for Health and Population

In reply please quote No.

....

MINISTRY OF HEALTH AND POPULATION
P.O. BOX 30377
LILONGWE 3
MALAWI

22nd July, 2018

Sekeleghe Kayuni
University of Liverpool
UK

Dear Sir,

Re: Protocol # 17/05/1805: Multidisciplinary Studies On Male Genital Schistosomiasis (Mgs): Its Prevalence, Morbidity And Management And Interactions With HIV Viral Shedding Among Adult Fisherman Along Lake Malawi Shores In Mangochi, Malawi

Thank you for the above titled proposal that you submitted to the National Health Sciences Research Committee (NHSRC) for review. Please be advised that the NHSRC has **reviewed** and **approved** your application for continuation of the above titled study.

- **APPROVAL NUMBER : 1805**
- The above details should be used on all correspondences, consent forms and documents as appropriate.
- **APPROVAL DATE : 23/07/2018**
- **EXPIRATION DATE**
  This approval expires on **22/07/2019**. After this date, this project may only continue upon renewal. For purposes of renewal, a progress report on a standard form obtainable from the NHSRC Secretariat should be submitted one month before the expiration date for continuing review.
- **SERIOUS ADVERSE EVENT REPORTING:** All serious problems having to do with subject safety must be reported to the NHSRC within 10 working days using standard forms obtainable from the NHSRC Secretariat.
- **MODIFICATIONS:** Prior NHSRC approval using forms obtainable from the NHSRC Secretariat is required before implementing any changes in the protocol (including changes in the consent documents). You may not use any other consent documents besides those approved by the NHSRC.
- **TERMINATION OF STUDY:** On termination of a study, a report has to be submitted to the NHSRC using standard forms obtainable from the NHSRC Secretariat.
- **QUESTIONS:** Please contact the NHSRC on phone number *+265 888 344 443* or by email on mohdoccentre@gmail.com.
- **OTHER:** Please be reminded to send in copies of your final research results for our records (Health Research Database).

Kind regards from the NHSRC Secretariat.

....

For: CHAIRPERSON, NATIONAL HEALTH SCIENCES RESEARCH COMMITTEE

SECRETARY FOR HEALTH
2018 -07- 22
P.O. BOX 30377, CAPITAL
LILONGWE 3

Promoting Ethical Conduct of Research[1]

Executive Committee: *Dr B. Chilima (Chairperson), Dr B. Ngwira (Vice-Chairperson)*
Registered with the USA Office for Human Research Protections (OHRP) as an International IRBIRB Number IRB00003905 FWA00005976
