# Supplementary material for: Detection of male genital schistosomiasis (MGS) by real-time TaqMan® PCR analysis of semen from fishermen along the southern shoreline of Lake Malawi
Source: Heliyon. 2023 Jun 21;9(7):e17338. doi: 10.1016/j.heliyon.2023.e17338 (PMC10394912; doi:10.1016/j.heliyon.2023.e17338)
Supplement: Multimedia component 5 [file mmc5.pdf]

INDIVIDUAL PARTICIPANT QUESTIONNAIRE

Research study on Male genital schistosomiasis and HIV among Adult fishermen living in Southern shores of Lake Malawi (MGS Baseline & Follow-up Survey)

Explanation: Put a tick (√) for the participant's responses appropriately. If the boxes are not enough on one page, use another sheet. Return this and other sheets to the Study Investigator at the end.

Date: (dd/mm/yy): __ __ / __ __ / __ __ Interviewer Name___________________________________

Consent checklist

Has written consent been obtained? Y ☐ N ☐ *Only proceed if Y*

Section A: Participant's details

1. Participant ID: ____
2. Village: ____
3. T/A: ____
4. Name of nearest Health centre: ________________________________
5. Gender: Male ☐ Female ☐.
6. Date of birth (dd/mm/yy): __ __ / __ __ / __ __ Age: ____________________
7. Born where: ____
8. How long have you been staying here: Years _____ Months: _____ Weeks: _____ Days: ______
9. Weight (kg): ____
10. Height (cm): ____

Section B: General Health Information

Are you experiencing the following symptoms now? In the last month? Put a tick **(√)** for **Yes** on all items mentioned or demonstrated:

	Symptom	Currently	In last month	Not in last month	Do not know	Refused
11.	Fever					
12.	Headache					
13.	Fatigue					
14.	Abdominal cramping (pain)					
15.	Pain during urination (dysuria)					
16.	Frequency of urination					
17.	Colour of urine					
18.	Blood in urine (haematuria)					
19.	Blood in stool					
20.	Blood in semen					
21.	Pains during and / or after coitus					
22.	Pains on ejaculation					
23.	Pains of the genital organs					
24.	Other (please specify):					

Are you experiencing the following diseases now? In the last month? Put a tick **(√)** for **Yes** on all items mentioned or demonstrated:

	**Disease**	**Currently**	**In last month**	**Not in last month**	**Don't know**	**Refused**
25.	Malaria					
26.	Diarrhoea					
27.	Dysentery					
28.	Skin disease					
29.	Respiratory disease					
30.	Worm infestation					
31.	Schistosomiasis					
32.	Sexually transmitted infection (STI)					
33.	Other (please specify):					

34. Did you take deworming medicine (albendazole) in the last month? Y ☐ N ☐.

35. Did you take schistosomiasis medicine (praziquantel) in the last month? Y ☐ N ☐.

36. Did you take antimalarial medicine in the last month? Y ☐ N ☐.

37. Did you take any other medicine in the last month? Y ☐ N ☐. If Y, name ________________

    ____

The following questions are about HIV/AIDS:

38. Did you attend any meetings about health education in the last month? Y ☐ N ☐.

39. Were any of the meetings about HIV/AIDS? Y ☐ N ☐.

40. Do you know what is HIV? Y ☐ N ☐.

41. Explain your answer in question 40.____

    ____

42. Do you know what is AIDS? Y ☐ N ☐.

43. Explain your answer in question 42.____

    ____

44. Do you know how HIV is transmitted? Y ☐ N ☐.

45. Explain your answer in question 44.____

    ____

46. Do you know how HIV is prevented? Y ☐ N ☐.

47. Explain your answer in question 46.____

    ____

48. Have you been tested for HIV? Y ☐ N ☐.

49. If Y in question 48, can you tell us the results? Y ☐ N ☐.

If Y in question 49, tell us the HIV test results? Tick **(√)** for the relevant answer below:

50. Positive (+) ☐ .
51. Negative (-) ☐ .
52. Do not remember ☐ .
53. If N in question 48, will you be willing to go for an HIV test? Y ☐ N ☐ .
54. If N in question 53, may you tell us the reason(s)?____

____

____

<u>Section C</u>: **<u>Hygiene and Sanitation Information</u>**

55. Do you swim, walk or work in the Lake? Y ☐ N ☐ . (*If N, go to question 57).*
56. If Y in question 55, how many times in a week?____
57. Do you bathe or wash in the Lake? Y ☐ N ☐ .
58. If Y in question 57, how many times in a week?____
59. If Y in question 57, why do you bathe or wash in the Lake? ________________________________

____

____

60. Do you wear shoes or long protective wear when in the Lake? Y ☐ N ☐ .
61. If Y in question 60, how many times when in the Lake?____________________________________
62. If N in question 60, why do you not wear them? ____

____

____

63. Where is the main place you go to urinate?____
64. Where is the main place you go to defecate?____
65. Does your house have a toilet? Y ☐ N ☐ .
66. If Y in question 65, do you use it? Y ☐ N ☐ .
67. If Y in question 66, how many times in a week?____
68. If N in question 66, why do you not use it? ____

____

____

69. If N in question 66, where do you use the toilet?____

____

Do you have access to treatments for schistosomiasis? Tick **(√)** for the relevant answer below:

70. No, not at all ☐ .

71. Yes ☐ .

72. Do not know ☐ .

73. Refused to answer ☐ .

Section D: Socio-economic characteristics

What is your highest level of education? Put a tick **(√)** for **Yes** on only one option:

	**Level of Education**	**Option**
74.	Never went to school	
75.	Not finished primary school (6 years)	
76.	Completed primary school	
77.	Not finished secondary school	
78.	Completed secondary school	
79.	Not finished tertiary/professional school	
80.	Completed tertiary/professional school	
81.	*Don't know*	
82.	*Refused*	

83. Do you have a job or are you employed? Y ☐ N ☐ .

If Y to question 83, can you tell us the job? Put a tick **(√)** for **Yes** on all items mentioned or demonstrated:

	**Type of Employment / Job**	**Yes**
84.	Self-employed. *Specify:*	
85.	Fishing	
86.	Farming/agriculture	
87.	Clerk/administration	
88.	Health worker	
89.	Selling at market	
90.	*Other. Specify:*	
91.	*Refused*	

Other employment status,

	**Other Job**	**Yes**
92.	Doing housework	
93.	Student	
94.	Retired	
95.	Long-term disabled	
96.	Unemployed	
97.	*Don't know*	
98.	*Refused*	

If Y to question 85 (fishing), can you tell us more about your work by answering the following:

99. How long hav you been doing this job? ____________________

100. How often do you go for fishing? ____________________

101. What kind of fish do you catch? ____________________

102. Is the job (fishing) seasonal? Y ☐ N ☐ .

103. If Y to question 102, when is the season? ____________________

104. If Y to question 102, what else do you do during off-season period? ____________________

____

105. Do you migrate to other places during / after fishing? Y ☐ N ☐ . ____________________

106. If Y to question 105, how often do you migrate in a year? ____________________

107. If Y to question 105, where do you migrate to? ____________________

108. If Y to question 105, why do you migrate? ____________________

____

____

109. Do you own a boat? Y ☐ N ☐ .

110. What kind of boat? ____________________

111. Are you planning of getting a new boat? Y ☐ N ☐ .

112. What kind of boat? ____________________

____

Regarding marriage and family,

Can you tell us about your relationship status:

	**Relationship**	**Yes**
113.	Married	
114.	Co-habiting / engaged	
115.	Divorced	
116.	Single	
117.	*Other. Specify:*	
118.	*Refused*	

119. Do you have children? Y ☐ N ☐ .

120. If Y to question 119, how many? ____________________

121. If N to question 119, why? ____________________

____

____

122. Has your spouse / partner experienced stillbirth / abortion? Y ☐ N ☐ .

123. Have you or your spouse / partner had childlessness after marriage/engaged? Y ☐ N ☐ .

124. Has you or your spouse / partner experienced infertility? Y ☐ N ☐ .

125. What do you think about pains of genital organs or during / after coitus if you experience it?

____

____

____

126. What do your spouse think about pains of your genital organs or during / after coitus? ______

____

____

____

Does your spouse or partner experience the following symptoms:

127. Lower abdominal pain? Y ☐ N ☐ . For how long ________________________________

128. Pain during / after coitus? Y ☐ N ☐ . For how long ______________________________

129. Bleeding during / after coitus? Y ☐ N ☐ . For how long ___________________________

130. Pain during menstruation? Y ☐ N ☐ . For how long ____________________________

131. Changes of menstrual flow / flow? Y ☐ N ☐ . For how long ______________________

132. If Y to any of the symptoms above, what does your spouse think about them? ____________

____

____

____

133. If Y to any of the symptoms above, what do you think about them? _____________________

____

____

____

134. Any other comments related to issues above? ______________________________________

____

____

____

THANK YOU FOR YOUR PARTICIPATION

INDIVIDUAL PARTICIPANT QUESTIONNAIRE

Research study on Male genital schistosomiasis and HIV among Adult fishermen living in Southern shores of Lake Malawi (MGS-HIV Baseline & Follow-up Survey)

Explanation: Put a tick (√) for the participant's responses appropriately. If the boxes are not enough on one page, use another sheet. Return this and other sheets to the Study Investigator at the end.

Date: (dd/mm/yy): __ __ / __ __ / __ __ Interviewer Name________________________________

Consent checklist

Has written consent been obtained? Y ☐ N ☐ *Only proceed if Y*

Section A: Participant's details

1. Participant ID: ____
2. Village: ____
3. T/A: ____
4. Name of nearest Health centre: ______________________________
5. Gender: Male ☐ Female ☐.
6. Date of birth (dd/mm/yy): __ __ / __ __ / __ __ Age: ____________________
7. Born where: ____
8. How long have you been staying here: Years _____ Months: _____ Weeks: _____ Days: ______
9. Weight (kg): ____
10. Height (cm): ____

Section B: General Health Information

Are you experiencing the following symptoms now? In the last month? Put a tick **(√)** for **Yes** on all items mentioned or demonstrated:

	Symptom	Currently	In last month	Not in last month	Do not know	Refused
11.	Fever					
12.	Headache					
13.	Fatigue					
14.	Abdominal cramping (pain)					
15.	Pain during urination (dysuria)					
16.	Frequency of urination					
17.	Colour of urine					
18.	Blood in urine (haematuria)					
19.	Blood in stool					
20.	Blood in semen					
21.	Pains during and / or after coitus					
22.	Pains on ejaculation					
23.	Pains of the genital organs					
24.	Other (please specify):					

Are you experiencing the following diseases now? In the last month? Put a tick **(√)** for **Yes** on all items mentioned or demonstrated:

	**Disease**	**Currently**	**In last month**	**Not in last month**	**Don't know**	**Refused**
25.	Malaria					
26.	Diarrhoea					
27.	Dysentery					
28.	Skin disease					
29.	Respiratory disease					
30.	Worm infestation					
31.	Schistosomiasis					
32.	Sexually transmitted infection (STI)					
33.	Other (please specify):					

34. Did you take deworming medicine (albendazole) in the last month? Y ☐ N ☐ .

35. Did you take schistosomiasis medicine (praziquantel) in the last month? Y ☐ N ☐ .

36. Did you take antimalarial medicine in the last month? Y ☐ N ☐ .

37. Did you take any other medicine in the last month? Y ☐ N ☐ . If Y, name _______________

____

The following questions are about HIV/AIDS:

38. Did you attend any meetings about health education in the last month? Y ☐ N ☐ .

39. Were any of the meetings about HIV/AIDS? Y ☐ N ☐ .

40. Have you experienced any problems after starting ART? Y ☐ N ☐ .

41. Did you had any problems also related to genitourinary organs in last 12 months? Y ☐ N ☐.

42. If Y in question 41, may you tell the problems?____

____

____

43. Did you seek any help on the problems said in question 42? Y ☐ N ☐ .

44. If Y in question 43, where did you get help?____

45. If Y in question 43, what help were you given?____

____

____

46. If N in question 43, may you tell the reason(s)?____

____

____

47. Will you accept to do a full clinical examination including the genitourinary system? Y ☐ N ☐ .

Section C: Hygiene and Sanitation Information

48. Did you swim, walk or work in the Lake? Y ☐ N ☐. *(If N, go to question 50).*
49. If Y in question 48, how many times in a week?____
50. Did you bathe or wash in the Lake? Y ☐ N ☐.
51. If Y in question 50, how many times in a week?____
52. If Y in question 50, why do you bathe or wash in the Lake? ________________________________
    ____
    ____
53. Do you wear shoes or long protective wear when in the Lake? Y ☐ N ☐.
54. If Y in question 53, how many times when in the Lake?____________________________________
55. If N in question 53, why do you not wear them? ____
    ____
    ____
56. Where is the main place you go to urinate?____
57. Where is the main place you go to defecate?____
58. Does your house have a toilet? Y ☐ N ☐.
59. If Y in question 58, do you use it? Y ☐ N ☐.
60. If Y in question 59, how many times in a week?____
61. If N in question 59, why do you not use it? ____
    ____
    ____
62. If N in question 59, where do you use the toilet?____
    ____

    Do you have access to treatments for schistosomiasis? Tick **(√)** for the relevant answer below:

63. No, not at all ☐ .
64. Yes ☐ .
65. Do not know ☐ .
66. Refused to answer ☐ .

Section D: **Socio-economic characteristics**

What is your highest level of education? Put a tick **(√)** for **Yes** on only one option:

	**Level of Education**	**Option**
67.	Never went to school	
68.	Not finished primary school (6 years)	
69.	Completed primary school	
70.	Not finished secondary school	
71.	Completed secondary school	
72.	Not finished tertiary/professional school	
73.	Completed tertiary/professional school	
74.	*Don't know*	
75.	*Refused*	

76. Do you have a job or are you employed? Y ☐ N ☐.

If Y to question 76, can you tell us the job? Put a tick **(√)** for **Yes** on all items mentioned or demonstrated:

	**Type of Employment / Job**	**Yes**
77.	Self-employed. *Specify:*	
78.	Fishing	
79.	Farming/agriculture	
80.	Clerk/administration	
81.	Health worker	
82.	Selling at market	
83.	*Other. Specify:*	
84.	*Refused*	

Other employment status,

	**Other Job**	**Yes**
85.	Doing housework	
86.	Student	
87.	Retired	
88.	Long-term disabled	
89.	Unemployed	
90.	*Don't know*	
91.	*Refused*	

If Y to question 78 (fishing), can you tell us more about your work by answering the following:

92. How long have you been doing this job? ____

93. How often do you go for fishing? ____

94. What kind of fish do you catch? ____

95. Is the job (fishing) seasonal? Y ☐ N ☐.

96. If Y to question 95, when is the season? _________________________

97. If Y to question 95, what else do you do during off-season period? _________________________

____

98. Do you migrate to other places during / after fishing? Y ☐ N ☐ .

99. If Y to question 98, how often do you migrate in a year? ____________________

100. If Y to question 98, where do you migrate to? ____________________

101. If Y to question 98, why do you migrate? ____________________

____

____

102. Do you own a boat? Y ☐ N ☐ .

103. What kind of boat? ____________________

104. Are you planning of getting a new boat? Y ☐ N ☐ .

105. What kind of boat? ____________________

____

Regarding marriage and family,

Can you tell us about your relationship status:

	**Relationship**	**Yes**
106.	Married	
107.	Co-habiting / engaged	
108.	Divorced	
109.	Single	
110.	*Other. Specify:*	
111.	*Refused*	

112. Do you have children? Y ☐ N ☐ .

113. If Y to question 112, how many? ____________________

114. If N to question 112, why? ____________________

____

____

115. Has your spouse / partner experienced stillbirth / abortion? Y ☐ N ☐ .

116. Have you or your spouse / partner had childlessness after marriage/engaged? Y ☐ N ☐ .

117. Has you or your spouse / partner experienced infertility? Y ☐ N ☐ .

118. What do you think about pains of genital organs or during / after coitus if you experience it?

____

____

____

119. What do your spouse think about pains of your genital organs or during / after coitus? ______

____

____

____

____

Does your spouse or partner experience the following symptoms:

120. Lower abdominal pain? Y ☐ N ☐. For how long ________________________________

121. Pain during / after coitus? Y ☐ N ☐. For how long _______________________________

122. Bleeding during / after coitus? Y ☐ N ☐. For how long ___________________________

123. Pain during menstruation? Y ☐ N ☐. For how long ______________________________

124. Changes of menstrual flow / flow? Y ☐ N ☐. For how long _______________________

125. If Y to any of the symptoms above, what does your spouse think about them? ____________

____

____

____

____

126. If Y to any of the symptoms above, what do you think about them? ____________________

____

____

____

____

127. Any other comments related to issues above? ______________________________________

____

____

____

THANK YOU FOR YOUR PARTICIPATION
