# Supplementary material for: Detection of male genital schistosomiasis (MGS) by real-time TaqMan® PCR analysis of semen from fishermen along the southern shoreline of Lake Malawi
Source: Heliyon. 2023 Jun 21;9(7):e17338. doi: 10.1016/j.heliyon.2023.e17338 (PMC10394912; doi:10.1016/j.heliyon.2023.e17338)
Supplement: Multimedia component 2 [file mmc2.pdf]

LSTM
LIVERPOOL SCHOOL OF TROPICAL MEDICINE
Pembroke Place,
Liverpool, L3 5QA, UK
Tel: +44(0)151 705 3100
Fax: +44(0)151 705 3370
www.lstmed.ac.uk

Dr Sekeleghe Kayuni
Liverpool School of Tropical Medicine
Pembroke Place
Liverpool
L3 5QA

Monday, 31 July 2017

Dear Dr Kayuni,

Re. Research Protocol (17-018) Multidisciplinary studies on Male Genital Schistosomiasis (MGS): Its prevalence, morbidity and management and interactions with HIV viral shedding among adult fisherman along Lake Malawi shores in Mangochi, Malawi

Thank you for your letter of 28 July 2017 providing the necessary in-country approvals for this project. I can confirm that the protocol now has formal ethical approval from the LSTM Research Ethics Committee.

The approval is for a fixed period of three years and will therefore expire on 30 July 2020. The Committee may suspend or withdraw ethical approval at any time if appropriate.

Approval is conditional upon:

- Continued adherence to all in-country ethical requirements.
- Notification of all amendments to the protocol for approval before implementation.
- Notification of when the project actually starts.
- Provision of an annual update to the Committee.
  Failure to do so could result in suspension of the study without further notice.
- Reporting of new information relevant to patient safety to the Committee
- Provision of Data Monitoring Committee reports (if applicable) to the Committee

Failure to comply with these requirements is a breach of the LSTM Research Code of Conduct and will result in withdrawal of approval and may lead to disciplinary action. The Committee would also like to receive copies of the final report once the study is completed. Please quote your Ethics Reference number with all correspondence.

Yours sincerely

Angela Obasi

Dr Angela Obasi
Chair
LSTM Research Ethics Committee

Researching and educating to save lives
A Company Limited by Guarantee. Registered Number 83405, England and Wales. Registered Charity Number 222655.

Athena SWAN Bronze Award
INVESTORS IN PEOPLE
